# Mapping regional cooperation of state actors for health research systems in Africa: A social network analysis

**DOI:** 10.1371/journal.pgph.0001142

**Published:** 2022-10-13

**Authors:** Aaron Hedquist, Catherine M. Jones, Rhona M. Mijumbi, Joëlle Sobngwi-Tambekou, Justin Parkhurst, Clare Wenham

**Affiliations:** 1 LSE Health, London School of Economics and Political Science, London, United Kingdom; 2 The Centre for Rapid Evidence Synthesis, College of Health Sciences, Makerere University, Kampala, Uganda; 3 Recherche-Santé & Développement (RSD Institute), Yaoundé, Cameroon; 4 Department of Health Policy, London School of Economics and Political Science, London, United Kingdom; PLOS: Public Library of Science, UNITED STATES

## Abstract

Regional bodies can potentially play an important role in improving health research in Africa. This study analyses the network of African state-based regional organisations for health research and assesses their potential relationship with national health research performance metrics. After cataloguing organisations and their membership, we conducted a social network analysis to determine key network attributes of national governments’ connections via regional organisations supporting functions of health research systems. This data was used to test the hypothesis that state actors with more connections to other actors via regional organisations would have higher levels of health research performance across indicators. With 21 unique regional organisations, the African continent is densely networked around health research systems issues. In general, the regional network for health research is inclusive. No single actor serves as a nexus. However, when statistics are grouped by African Union regions, influential poles emerge, with the most predominate spheres of influence in Eastern and Western Africa. Further, when connectivity data was analysed against national health research performance, there were no statistically significant relationships between increased connectivity and higher performance of key health research metrics. The inclusive and dense network dynamics of African regional organisations for health research strengthening present key opportunities for knowledge diffusion and cooperation to improve research capacity on the continent. Further reflection is needed on appropriate and meaningful ways to assess the role of regionalism and evaluate the influence of regional organisations in strengthening health research systems in Africa.

## Introduction

Regional organisations are increasingly involved in health policy matters within their broader sectoral integration mandates, [1] including in Africa [2, 3]. Public health emergencies such as the 2014 Ebola outbreak and current COVID-19 pandemic have highlighted the unique roles that regional organisations in Africa can play in coordination, resource pooling, or scientific leadership when they have institutional capacity for these [4–6]. The involvement of regional organisations in epidemics and disease control has been identified as a way to strengthen regional cooperation in health within Africa through diplomacy, with such organisations seen to be operating at the interface of global health institutions and national governments—seeking to represent and act on their shared interests, align strategies, promote a unified position, or mobilise partnerships [3, 7, 8].

Although COVID-19 analysis has reminded us of the role of regional organisations in Africa to support member states’ preparedness and response, some regional organisations, like the West African Health Organisation, have reported building on their experience from previous epidemics and their work to strengthen health research capacity across member states [9, 10]. To advance a regional approach to research capacity strengthening, cross-border collaboration between government agencies, research institutions and researchers has arisen from these emergencies, as in the instance of the establishment of the West Africa Consortium for Clinical Research on Epidemic Pathogens formed since the Ebola virus disease outbreak of 2014–2015. Health research capacity within national health research systems is fundamental to produce and use knowledge that informs national preparedness and response to health emergencies [11–13].

But the urgency and significance of an agenda to strengthen research capacity in African countries is not novel. The landmark report of the Commission on Health Research and Development in 1990 [14] argued that local research systems and capacity are essential to reduce health inequities and advance knowledge in and for low- and lower-middle income countries. International organisations and funding agencies have promoted health research capacity strengthening through partnerships and initiatives, which have produced and supported numerous collaborations and mechanisms to advance this agenda. For example, the European and Developing Countries Clinical Trials Partnership [15], the Sub-Saharan African Network for TB/HIV Research Excellence (SANTHE) [16], the African Institutions Initiative [17], the African Doctoral Dissertation Research Fellowship (ADDRF) program [18], the African Network for Drugs and Diagnostics Innovation (ANDI) [19], the WHO Special Programme for Research and Training in Tropical Diseases (TDR) [20, 21], and the Consortium for Advanced Research Training in Africa (CARTA) [22, 23]–to name a few—have demonstrated research capacity improvements at various levels. The core message across this body of knowledge, including from our research on nine national health research systems in Africa [24], is that leadership and ownership of partnerships by African research leaders, institutions, or governments are vital for sustainable, independent research capacity. While partnerships, collaborative strategies, and consortia between external and African partners have demonstrated great potential for reinforcing human and institutional resources for health research, power asymmetries and inequitable relationships between partners remain key issues [25–28].

Survey studies by African researchers in collaboration with the WHO Regional Office for Africa have shown general improvement in strengthening national health research systems over the past two decades on the aggregate level, based on data collected from national health research focal points and the literature [29–33]. Knowledge from evaluations and case studies on national health research system strengthening provide insight into issues like infrastructure, training, and political will [34–40], which, with issues of adequate financing, have persisted across time although systems and contexts vary [41, 42]. Yet, investment in health research in Africa remains inadequate to meet these needs, and there are disparities in health research capacity within and between the five regions of the continent [43, 44].

Regional organisations offer one route through which regional cooperation may extend to health research and potentially contribute to reducing differences in national health research capacity. Our previous qualitative work with national health science research decision-makers in Africa found the role of regional bodies (which have pre-existing cooperation structures, mechanisms, and processes) have been underexplored. Health, science, and higher-education decision-makers in government shared that they saw opportunities for using regional organisations to include health sciences research as a domain of cooperation (where there were not already dedicated regional bodies for this such as in Western and Eastern Africa) [24]. In a more recent qualitative study, we found that regional organisations in Africa are more involved in governance and research use and dissemination, than financing or infrastructure development–with capacity strengthening activities more focused on individual human resources than institutions [45]. Some regional organisations have policies specific to health research, such as the African Union Development Agency’s *Health Research and Innovation Strategy for Africa (2018–2030)* [46] and the WHO’s Regional Office for Africa *Research for Health Strategy for the African Region (2016–2025)* [47]. The mandates of regional organisations influence their involvement however, and many informants identified gaps in regional organisations’ involvement in coordination, infrastructure, and advocacy for strengthening health research systems [45]. Health research regionalism could foster interdependence to harness knowledge and capacity in member states, and coordinate resources, information, and materials to benefit national health research systems more equitably.

Acknowledging this history of regional bodies on the African continent and their health programmes and policies [2, 3, 48–50], with emerging knowledge about their roles in health research [5, 39, 45], there is still much unknown about the constellation of actors in regionalism for health sciences research (HSciR). Therefore, this study aims to map the state-based regional organisations in Africa involved in HSciR, characterise the network of actors, and test the hypothesis of whether regional network strength correlates with national health research indicators. By exploring the nature of the network of state actors interacting in regional bodies, the paper visualises this landscape and identifies key strengths, limitations, and implications of these interactions for regional cooperation to strengthen health research.

## Methods

We define regional organisations as state-based membership organisations with a specific geographic mandate for at least one of the five African regions as defined by the African Union (AU) [51] or language mandate. This definition of regional organisations adopts a “state-centric perspective” of regional cooperation from international relations [52]. We included organisations with direct or indirect interests in health research, health, or health systems. This includes regional economic communities (RECs) and specialist regional organisations in health or related sectors like higher education; science; and development.

We developed an initial list of regional organisations informed by interviews with national health sciences research stakeholders from nine African countries conducted within a previous research project [24]. We reviewed organisations’ websites, governing documents, policies, and strategic plans available online to assess evidence of their interest or involvement in strengthening at least one domain of health research systems [53] and to identify other relevant regional bodies operating on the continent. This process was repeated for identified organisations until no new bodies were found. Finally, external partners and networks with expertise in regional health cooperation and health research in Africa corroborated this list. The list of stakeholders is available in S1 File.

Focusing on African-led regionalism, the network analysis excluded international organisations with a regional presence in Africa, even if they are active in health and have African governance structures. International organisations, like the WHO Regional Office for Africa (WHO AFRO), have well-documented involvement and influence on African health research agendas and capacity as evidenced by the ongoing studies by WHO AFRO using data from the African Barometer collected from health research focal points in country [32] or similar studies with the WHO Regional Office for the Eastern Mediterranean (WHO EMRO) [54, 55]. Our qualitative research with informants in regional organisations confirms and elaborates on the important roles played by these two WHO regional offices [45]. However, we excluded United Nations specialised technical agencies to highlight African-initiated and owned regional institutions. The governance structures and interactions of international organisations with regional offices extend beyond African states and therefore, their inclusion in our analysis would inhibit our ability to visualize and understand uniquely African-led state membership networks.

We designed a Social Network Analysis (SNA) to measure the number of connections between African states through regional organisations related to health research. The SNA privileged sub-organisations of continental groups with more specific geographic mandates over secretariats to better capture regional networks. We elected for this inclusion and exclusion criteria to clearly delineate between continental organisations (i.e., bodies that represent the entirety, or large part, of the African continent) and regional organisations (i.e., bodies with membership of geographically grouped countries). Therefore, in lieu of including continental organisations (e.g., Africa CDC or the African Development Bank), we elected to include their regional networks or counterparts (e.g., the five Africa CDC Regional Collaborating Centres or the five African Development Bank Regional Integration Offices). By doing so, the SNA is better powered to focus on the unique contours of African regions as defined by the AU [51].

An adjacency matrix was created for each included organisation, extracting data on member states of each organisation included in the stakeholder list (see S2 File). The individual country was designated the node with edges representing connections via regional organisations. Weighted degrees (or the number of connections to and from a country) were calculated by summing each matrix. We designed this weighting system to highlight countries that connect multiple times through different organisations.

Network data was analysed using the open-source SNA software, *Gephi (version 0*.*9*.*2)* with additional analysis to determine standard deviations and group statistics by region [56]. This SNA focused on eigenvector centrality (EC)—a relative measure of network influence that benefits countries with more connections to other highly connected countries. The mean EC for each region were also calculated to identify those with a higher concentration of centrally connected countries. Network density (the number of observed country connections as a proportion of the total possible connections) and network diameter (the number of connections between the two most distant nodes) were calculated to describe the network. We drew definitions and guidance from general sources on the SNA method and sources specific to SNA in health policy and systems research [57, 58].

The results of the SNA were used to test a hypothesis that increased membership to regional organisations for health or health research would be correlated with higher health sciences research performance metrics. Informants from our previous research highlighted that regional organisations may facilitate health research collaboration, information sharing, and knowledge dissemination [24]. Therefore, we selected four indicators based on the potential that they might be associated with regionalism collaboration: gross domestic expenditure on R&D (GERD) as a percentage of gross domestic product (GERD/GDP); clinical trials per million population (TRIALS); patent applications per million population (PATENTS); and researchers per million population (RESEARCHERS). The data set used was from a previous study on metrics of African health sciences research capacity [44]. The hypothesis was tested using a Pearson’s r test to determine correlation coefficients, with a significance level set at p = 0.05.

### Strengths and limitations

The analysis did not weigh regional organisations by subject matter or relative influence. This means that regional organisations with high policy area relevancy, like the West African Health Organisation, the East African Health Research Commission, or the *Organisation de Coordination pour la Lutte contre les Endémies en Afrique Centrale* were counted the same as organisations with less direct connection to health, like the Indian Ocean Commission, the Arab Maghreb Union, or the Community of Sahel-Saharan States. This could reduce the SNA effect size as regions that engage more comprehensively through a smaller number of specialised organisations would not be emphasised. Ideally, we might weigh this analysis by organisation-level HSciR-related expenditures, however, this information is not readily available to the public. Future analysis would benefit from more financial transparency from regional organisations.

Our focus on regional cooperation for health research between African states excluded private and public-private partnership organisations. Thus, our SNA of regional stakeholders does not capture countries’ network connectivity through private and hybrid organisations nor their potential effects. For example, in our initial landscape review, we identified several private organisations and NGOs active in the HSciR space. These organisations are likely strong contributors to regional HSciR efforts. Also, due to our focus on an African state-centric regional cooperation, relationships and collaborations with donors and external partners are not included. While this may be seen as a limitation to the analysis given the well-documented contributions of international collaborations and investment to support research capacity strengthening, the purpose of this SNA to centre state actors in collaboration builds on our findings related to the coordination and advocacy gaps identified by regional organisations for their roles in strengthening health research systems [45]. The scope of this SNA provides a picture of opportunities for inter-state regional cooperation on HSciR development using existing regional cooperation structures and not an analysis of all networks operating in the research capacity strengthening space in Africa. That said, the inclusion of regional organisations that are not RECs or regional health (sector) organisations but have direct implications and involvement in areas of health research systems is a key strength of our method (e.g. the African Regional Intellectual Property Organization and the *Conseil Africain et Malgache pour l’Enseignement Supérieur*). Given the heterogeneity of subject matter included in our SNA, the expansion of the inclusion criteria to include non-state-based and external actors would limit interpretability for state-based networks.

Availability and missing data for metrics of health sciences research capacity are additional limitations for interpreting the insignificant association between regional organisation membership and national performance. The dataset was comprised of the most up-to-date statistics as of 2017, or closest available year (e.g., 2016 for patents) [44]. While most regional organisations were established much earlier, some organisations were founded in the 2010s. For example, the Africa CDC was established the same year many of the included metrics were collected. Therefore, the potential impact of these younger organisations would not be represented in this analysis. Thus, the implications of the Pearson test should be interpreted with caution, as a first step to pilot such as assessment.

## Results

In total, this study identified 21 unique state-based membership regional organisations that are either directly or indirectly involved in health research policy or strengthening activities in Africa. Among these, 26 relevant sub-organisations were identified with either more specific geographic mandate or additional policy area specialisation. By sector, health organisations (n = 17) were the most represented, amongst representation from economic (n = 11), development (n = 8), science (n = 7), political (n = 2), and higher education organisations (n = 2). Overall, 34 organisations were included in the SNA with the full list available in S2 File. Below we present the results on the connectivity of states and the characteristics of regional networks, and the analysis of the relationship between regional connectivity and national health research capacity. The full country level SNA summary is available in S3 File.

**Fig 1:**
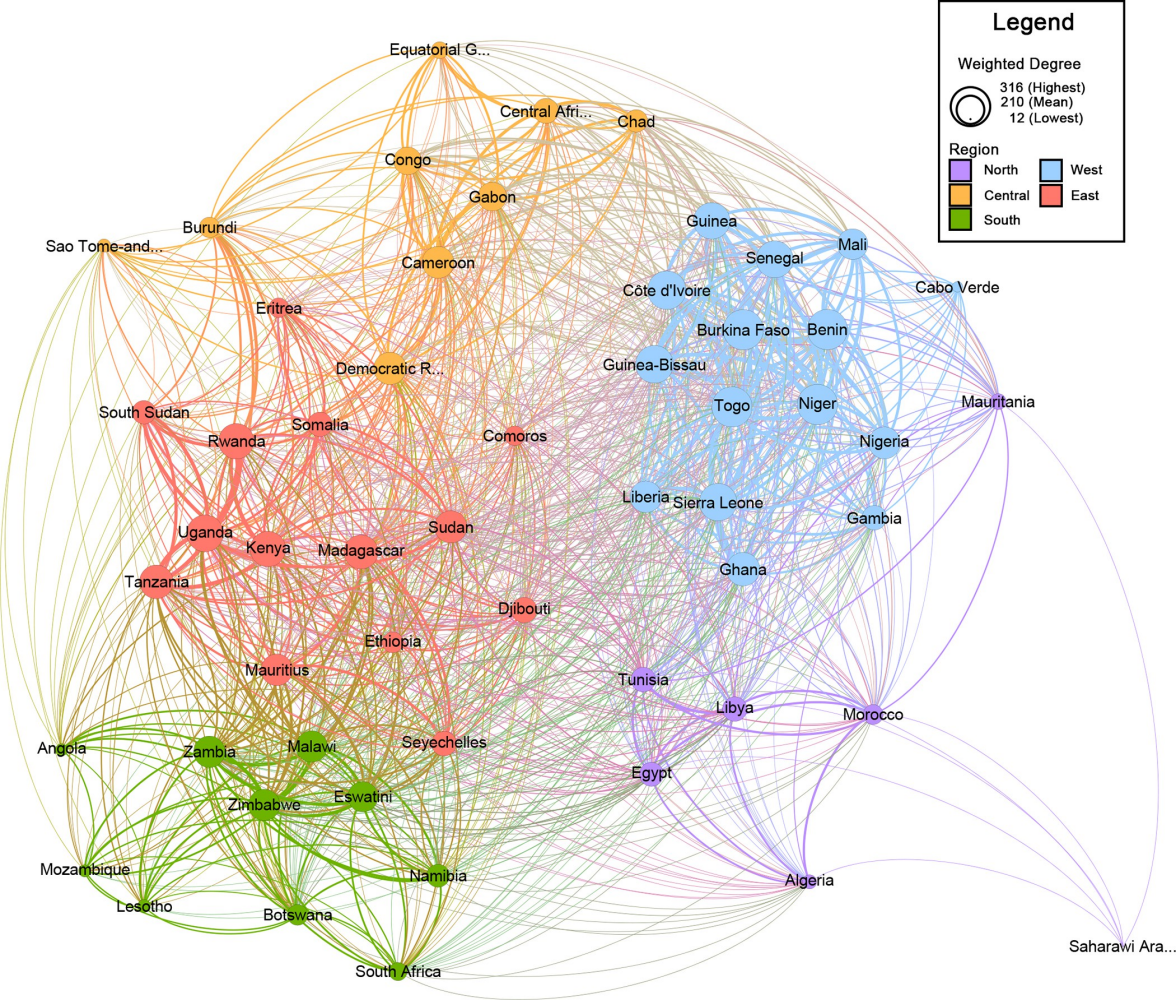
African state-based network map for regional HSciR. Legend: States are represented by individual nodes and connections between states represented by edges. More frequent connections are reflected with thicker edges. The total weighted degree for each State is indicated by the node size.

We found that African countries are tightly networked around health research in regional organisations with nearly 80% of possible connections between countries currently present (network density of 0.795). This dense network is complemented by a small network diameter of three, which indicates that information can theoretically spread efficiently throughout the network and no country is entirely excluded. These results are illustrated in Fig 1 with descriptive statistics in Table 1.

**Table 1:** Summary table of SNA statistical measures.

		Weighted Out-Degree	Average Per Country (SD)	Mean Eigenvector Centrality (SD)
**Central Africa**	**Central Africa Total**	**858**	**95 (24.5)**	0.81 (0.16)
	Central Africa	328	36 (8.0)	
	Eastern Africa	213	24 (10.9)	
	Northern Africa	47	5 (3.2)	
	Southern Africa	63	7 (6.2)	
	Western Africa	207	23 (10.0)	
**Eastern Africa**	**Eastern Africa Total**	**1560**	**111 (24.1)**	0.94 (0.04)
	Central Africa	213	15 (6.1)	
	Eastern Africa	714	51 (13.6)	
	Northern Africa	117	8 (2.2)	
	Southern Africa	278	20 (10.3)	
	Western Africa	238	17 (4.7)	
**Northern Africa**	**Northern Africa Total**	**487**	**70 (30.2)**	0.77 (0.29)
	Central Africa	47	7 (10.8)	
	Eastern Africa	117	17 (6.3)	
	Northern Africa	134	19 (4.6)	
	Southern Africa	47	7 (10.2)	
	Western Africa	142	20 (3.1)	
**Southern Africa**	**Southern Africa Total**	**876**	**88 (30.8)**	0.78 (0.20)
	Central Africa	63	6 (12.9)	
	Eastern Africa	278	28 (3.3)	
	Northern Africa	47	5 (8.4)	
	Southern Africa	372	37 (6.1)	
	Western Africa	116	12 (2.5)	
**Western Africa**	**Western Africa Total**	**2003**	**134 (30.2)**	0.88 (0.18)
	Central Africa	207	14 (5.7)	
	Eastern Africa	238	16 (3.4)	
	Northern Africa	142	9 (4.3)	
	Southern Africa	116	8 (15.5)	
	Western Africa	1300	87 (7.7)	
**Total Weighted Out-Degree**		**5784**	**105 (35.6)**	
**Network Mean Eigenvector Centrality (SD)**		**0.85 (0.19)**		
**Network Density**		**0.795**		
**Network Diameter**		**3**		

This dense network is further evidenced by a high EC score across countries (mean = 0.85±0.19). This distribution indicates that most countries hold well connected, central positions in the network. However, there are connective outliers such as the Sahrawi Arab Democratic Republic (EC = 0.12). The country’s unique political status, being recognised by the AU but not the United Nations, could explain limited connections with other countries.

While no individual country stands out as uniquely central, when SNA statistics are grouped by AU region, Western and Eastern Africa are better networked for health research and have a higher distribution of well-connected countries compared to other regions. This SNA grouped two statistics by region, weighted out-degrees (the number of single direction connections from country X to country Y, excluding reciprocal connections from country Y to country X), and EC. Western Africa is the most networked region with an average 134±30.2 connections per country. The majority of these connections are to other West African countries (mean = 87±7.7). This is a striking result, as the region’s internal connectivity outsizes all other regions. In Northern Africa, for example, each country only maintains an average 19±4.6 degrees of connection within other North African countries.

Eastern Africa has the second highest number of connections, with an average of 111±24.1 connections per country. In comparison to Western Africa, less than half of Eastern Africa’s connections are to other East African countries (mean = 51±13.6). This means that Eastern Africa maintains a looser internal network, especially among peripheral countries (mostly, the island-nations) but has stronger connections, proportionally, with the rest of the continent than Western Africa.

Being more networked, Western and Eastern Africa also maintain more central and therefore, potentially influential positions in the continental network. Western Africa and Eastern Africa had the highest mean EC values of any region (0.88±0.18; 0.94±0.04 respectively). This could be explained by the presence of more specialist sub-organisations for health and health research within their regional economic communities, such as the West African Health Organisation and East African Health Research Commission. Also, these two regions have the greatest number of countries of all AU regions, which would amplify their impact in the network. Northern Africa maintained the least central position in the network with a mean EC of 0.77±0.29, followed closely by Southern Africa (0.78±0.20).

Considering the dense network and regional poles, we explore the possible associations between connectivity through regional organisations and national health research capacity. We produced correlation coefficients between weighted degree (mean = 210.32±71.21), or the number of connections to and from a country, and four health science research metrics—GERD per GDP (mean = 0.35±0.24), researchers per million population (mean = 193.15±380.67), clinical trials per million population (mean = 11.64±15.67), and patent applications per million population (mean = 4.67±10.87).

GERD/GDP and TRIALS were not correlated with weighted degree. We observed a small negative correlation between RESEACHERS and weighted degree with a correlation coefficient of r(33) = -0.16, p = 0.3626. The strongest relationship was a negative correlation between PATENTS (r(21) = -0.36, p = 0.08915). Without achieving statistical significance for any of these coefficients, the results of this initial assessment reject the hypothesis that increased connection through regional organisations is associated with better national health science research metrics. The summary table of results is included in Table 2.

**Table 2:** Summary table of results for Pearson-R test of weighted degree and health science research metrics.

Variable	Degrees of Freedom	Mean (SD)	Correlation Coefficient (P Value)
Weighted Degree		210.32 (71.21)	
GERD Per GDP	29	0.35 (0.24)	0.04 (p = 0.8238)
Researchers per Million Population	33	193.15 (380.67)	-0.16 (p = 0.3626)
Clinical Trials per Million Population	52	11.64 (15.67)	-0.05 (p = 0.7014)
Patent Applications per Million Population	21	4.67 (10.87)	-0.36 (p = 0.08915)

## Discussion

The results from this SNA present an optimistic case for African regionalism for health research despite statistically insignificant results about its relationship to national health research performance. With 21 unique organisations and an additional 26 specialised sub-organisations, the African continent houses a vast and dense network of regional bodies related to health research systems. Our results show that governments are densely networked through regional cooperation with very few states being left on the periphery. These findings support claims that the design of African state-based regional networks for cooperation in matters of health research systems is suited for efficient communication between state actors. This is an important asset to national health research systems, as, theoretically, lessons learned in one locality could easily be shared throughout the network either in continental forums or in more specialised regional discussions. One key challenge to this is that regional organisations tend to be organised by policy sector [45], while health research systems are multi-sectoral [24, 59, 60]. This means that the health, science and innovation, and higher education sectors need to coordinate at the national level to incorporate such information coming through state representatives via respective ministries or departments to various regional organisations [24, 33].

However, without significant correlations between increased regional connections and improved national health research performance, the benefits of African regionalism for health research are inconclusive and merit further exploration. For instance, a critical perspective on the selection and use of global metrics to measure performance of health research systems in Africa is needed, which we have reflected upon elsewhere [61]. The HSciR performance metrics which have been developed, defined, collected, and disseminated for the most part by institutions in the global north have important consequences when they are recommended or imposed as indicators for use in decision-making and monitoring health research systems in other contexts; there are several research and collaboration processes related to power, equity, and ownership hidden behind and within these numbers [61]. Despite methodological, ethical, political and practical issues with HSciR performance evaluation and use of these metrics for African institutions [61–63], these are among the core set of indicators used to track, benchmark, and analyse HSciR development and performance at a global level in the [64–66]. In our work mapping available indicators for HSciR in all 54 African countries, we found a nuanced picture, whereby health research systems performed differently across indicators using the state jurisdiction as the unit of analysis (national level) [44], a finding which has also been shown in studies using indicators of knowledge economies in Africa [67]. Through the lens of a national jurisdiction, countries do not perform homogenously across metrics, showing different areas of strengths influenced by several factors. The snapshot of performance may look different if using a regional or institutional level of analysis.

Nevertheless, within these limitations, the SNA results point to key opportunities to use state actor-based regional cooperative networks in strategic and targeted ways. Most prominently, some higher-performing countries in health research are not central or deeply engaged in the broader network. South Africa, for example, performs well on key health research metrics like the number of clinical trials per capita and GERD per GDP [44]. It also maintains world-class universities, houses the only vaccine manufacturer in sub-Saharan Africa, and controls 70% of sub-Saharan pharmaceutical production [68]. Yet, South Africa’s engagement with the regional cooperation system is in the lowest quintile. Given the dense system dynamics, further engagement from South Africa and other states with higher performing national health research systems could benefit neighbouring regional countries.

Beyond individual countries’ connections, the structures of regional cooperation reflect the history and consequences of colonial exploitation. Western Africa’s position as an outlier with strong intra-regional connectivity for health research could be rooted in its pattern of regional economic integration more broadly as an approach to support development, peace and security. Some argue that regional cooperation was an imperative upon independence, not least for economic and political reasons, but also to bridge populations and culture towards shared identity and governance across borders imposed by French and British colonists [69, 70]. Cooperation between West African states also built on a history of dense commercial, social, and political networks before colonisation [70]. Francophone countries in Western Africa maintained regional organisations prior to the establishment of the Economic Community of West African states in 1975 [69]—evidenced by the *Organisation de Coordination et de Coopération pour la Lutte Contre les Grandes Endémies* (est.1960), which merged with the Anglophone West African Health Community (est. 1972) to form the West African Health Organisation in 1987.

In contrast, Northern Africa’s weaker position in the continental network could also be explained by historical ties with the Middle East. Nonetheless, North African countries offer useful lessons for building health-related industries such as Egypt’s predominate generic manufacturing capacities [71] and for strengthening the legal framework for health research and investment in innovation such as Tunisia’s law on scientific research orientation [24]. Encouraging and facilitating North African countries to develop a more connected position in African regionalism for health research could bolster opportunities for learning, networking, and potentially increased performance across the region, and indeed continent.

National decision-makers’ awareness and appreciation of health research is a challenge for high-level commitments to strengthen health research systems domestically and through regional organisations, which is why advocacy is key to influencing political will and interest [45]. But as national research leaders and other advocates make the case for prioritising health research in their countries, the incentives may be less clear for states with stronger research capacity or higher performance to engage with regional organisations for this even if senior civils servants have expressed interest in more inter-regional cooperation. Therefore, for regionalism for HSciR to prosper, health researchers, leaders, and regulators need to advocate to government and state representatives in regional organisations on the opportunities and benefits of using regional organisations as networks for strengthening national health research systems through learning, exchanging, and cooperating.

### Future areas of study

In this investigation, increased connectivity was not shown to produce meaningful effect when assessed with basic tests of association at a single time point. With this, the null hypothesis must be explored further. Deeper analysis of longitudinal trends at the national or continental levels could provide key insights into the strengths and limitations of regional bodies. However, it was quite difficult to validate inception dates for many regional organisations. In some instances, regional organisations that are currently active today were founded out of previous organisations. Therefore, without careful consideration at the initial design of the study, it would be difficult to create a validated longitudinal dataset.

We elected not to pursue additional hypothesis testing after the initial conclusion as HSciR metrics are difficult to validate with publicly available information, especially as there are no uniform data collection or reporting standards across the continent [44]. We believe further research that engages critically with HSciR metrics could provide insights into the potential role of state-based cooperation networks for national health research systems.

There is an underlying question about whether assessing a regional organisation by performance indicators at the national level is the most effective way to capture effect. Aggregating health research indicators to the regional level is one possibility, but this would reproduce issues with data availability and appropriateness. Indeed, the relevance and use of standard international metrics for evaluating health research performance in African countries is not without critique and limitations as well [61]. Another option would be to review health research policy diffusion or research uptake in a regional network as a measure of cooperation effects. For example, research has shown that African RECs have incorporated health to varying degrees into their policy portfolios, but there is still a lack of knowledge about their impacts on national health or health research systems [2]. Measuring the impact of regional cooperation should also account for challenges of multi-level governance [45].

## Conclusion

In this effort to characterise the African network of state-based regional cooperation for health research systems, we confirmed that a dense and inclusive network of countries exists through regional organisations. These results provide an encouraging view of intra- and inter-regional connectivity among state actors. However, evaluating the impact of regional cooperation on strengthening health research and identifying mechanisms to increase interaction of Northern and Southern Africa in the network are core issues to be addressed in African regionalism for health research improvement. As these findings join a growing body of literature on health regionalism in Africa, we think they underscore the importance of research needed to better understand the processes, outcomes, and impacts of south-south state cooperation at the regional level on policies, systems, and capacity for health research.

## Supporting information

S1 File Stakeholder list. A list of African regional organisations identified during review.
(DOCX)

S2 File Social network analysis dataset. The underlying dataset that was used to generate the social network analysis.
(XLSX)

S3 File Social network analysis country-level summary statistics. A summary table of the social network analysis statistical results combined with country-level health science research metric outcomes.
(DOCX)
