# Supplementary material for: Mapping regional cooperation of state actors for health research systems in Africa: A social network analysis
Source: PLOS Glob Public Health. 2022 Oct 13;2(10):e0001142. doi: 10.1371/journal.pgph.0001142 (PMC10022136; doi:10.1371/journal.pgph.0001142)
Supplement: S1 File — A list of African regional organisations identified during review. (DOCX) [file pgph.0001142.s001.docx]

**S1 File: Stakeholder List**

| Policy Area | Primary Organisation | Sub Organisation | Included in SNA |
| --- | --- | --- | --- |
| Health | Africa CDC | Secretariat | No |
| Health | Africa CDC | Central Africa Regional Collaborating Centre | Yes |
| Health | Africa CDC | Eastern Africa Regional Collaborating Centre | Yes |
| Health | Africa CDC | Northern Africa Regional Collaborating Centre | Yes |
| Health | Africa CDC | Southern Africa Regional Collaborating Centre | Yes |
| Health | Africa CDC | Western Africa Regional Collaborating Centre | Yes |
| Development | African Development Bank | Secretariat | No |
| Development | African Development Bank | Central Regional Integration Office | Yes |
| Development | African Development Bank | East Regional Integration Office | Yes |
| Development | African Development Bank | North Regional Integration Office | Yes |
| Development | African Development Bank | South Regional Integration Office | Yes |
| Development | African Development Bank | West Regional Integration Office | Yes |
| Economic | African Organisation for Standardisation | Secretariat | Yes |
| Science | African Regional Intellectual Property Organization | Secretariat | Yes |
| Science | African Union | African Observatory of Science Technology and Innovation | No |
| Science | African Union | African Scientific, Research and Innovation Council | No |
| Science | African Union | Specialised Technical Committee on Health, Population, and Drug Control | No |
| Political | African Union | Commission | No |
| Science | African Union Development Agency | African Ministerial Council on Science and Technology | No |
| Health | African Union Development Agency | Southern African Network for Biosciences | Yes |
| Health | African Union Development Agency | Biosciences Eastern and Central Africa | Yes |
| Health | African Union Development Agency | West African Network of Biosciences | Yes |
| Health | African Union Development Agency | North African Network for Biosciences | Yes |
| Development | African Union Development Agency | Secretariat | No |
| Health | African Union Development Agency | African Medicines Regulatory Harmonization Initiative | No |
| Science | African Union Development Agency | Center of Excellence: Science, Technology and Innovation Hub | No |
| Economic | Arab Maghreb Union | Secretariat | Yes |
| Economic | Common Market for Eastern and Southern Africa | Secretariat | Yes |
| Economic | Community of Sahel-Saharan States | Secretariat | Yes |
| Education | Conseil Africain et Malgache pour l’Enseignement Supérieur | Secretariat | Yes |
| Economic | East African Community | Secretariat | Yes |
| Science | East African Community | Science, Technology and Innovation Commission | Yes |
| Health | East African Community | East African Health Research Commission | Yes |
| Education | East African Community | Inter-University Council for East Africa | Yes |
| Health | East, Central, and Southern Africa Health Community | Secretariat | Yes |
| Economic | Economic Community of Central African States | Secretariat | Yes |
| Economic | Economic Community of West African States | Secretariat | Yes |
| Health | Economic Community of West African States | West African Health Organisation | Yes |
| Political | Indian Ocean Commission | Secretariat | Yes |
| Economic | Intergovernmental Authority on Development | Secretariat | Yes |
| Economic | Mano River Union | Secretariat | Yes |
| Economic | Organisation Africaine de la Propriété Intellectuelle | Secretariat | Yes |
| Health | Organisation de Coordination pour la Lutte contre les Endémies en Afrique Centrale | Secretariat | Yes |
| Economic | Southern African Development Community | Secretariat | Yes |
| Development | United Nations Economic Commission for Africa | Secretariat | No |
| Health | World Health Organisation | African Regional Office | No |
| Health | World Health Organisation | Eastern Mediterranean Regional Office | No |
