# Supplementary material for: Mapping regional cooperation of state actors for health research systems in Africa: A social network analysis
Source: PLOS Glob Public Health. 2022 Oct 13;2(10):e0001142. doi: 10.1371/journal.pgph.0001142 (PMC10022136; doi:10.1371/journal.pgph.0001142)
Supplement: S3 File — A summary table of the social network analysis statistical results combined with country-level health science research metric outcomes. (DOCX) [file pgph.0001142.s003.docx]

***S3 File. Social Network Analysis Country-Level Summary Statistics***

| Country | Region | Weighted Degree | Degree | Eigencentrality | Trials per Million | Patent Applications per Million | Researchers per Million | GERD Per GDP |
| --- | --- | --- | --- | --- | --- | --- | --- | --- |
| Algeria | North | 110 | 41 | 0.831601 | 4.06 | 2.61 | 168.02 | 0.07 |
| Angola | South | 96 | 24 | 0.452893 | 0.56 |  | 47.48 |  |
| Benin | West | 316 | 49 | 0.971811 | 4.97 |  |  |  |
| Botswana | South | 162 | 45 | 0.902957 | 41.33 | 0.44 | 179.47 | 0.54 |
| Burkina Faso | West | 316 | 49 | 0.971811 | 9.98 | 0.11 | 47.58 | 0.22 |
| Burundi | Central | 160 | 39 | 0.779562 | 1.81 |  |  | 0.12 |
| Cabo Verde | West | 82 | 14 | 0.287713 | 0 |  | 49.21 | 0.07 |
| Cameroon | Central | 250 | 49 | 0.971502 | 4.82 |  |  |  |
| Central African Republic | Central | 196 | 38 | 0.752394 | 2.83 |  |  |  |
| Chad | Central | 176 | 35 | 0.68841 | 0.83 |  | 58.33 | 0.32 |
| Comoros | East | 154 | 48 | 0.93971 | 3.77 |  |  |  |
| Congo | Central | 218 | 49 | 0.971502 | 10.14 |  | 31.54 |  |
| Cote d'Ivoire | West | 302 | 49 | 0.971811 | 3.04 | 1.1 | 69.21 |  |
| Democratic Republic of Congo | Central | 254 | 50 | 0.979794 | 1.1 |  | 7.23 | 0.02 |
| Djibouti | East | 200 | 47 | 0.952987 | 4.24 | 1.06 |  |  |
| Egypt | North | 188 | 48 | 0.955689 | 38.78 | 9.59 | 680.3 | 0.71 |
| Equatorial Guinea | Central | 132 | 29 | 0.569374 | 4.91 |  |  |  |
| Eritrea | East | 154 | 43 | 0.862764 | 1.34 |  |  |  |
| Eswatini | South | 236 | 47 | 0.938645 | 16.38 |  | 119.14 | 0.27 |
| Ethiopia | East | 164 | 42 | 0.877216 | 1.84 | 0.12 | 44.97 | 0.6 |
| Gabon | Central | 226 | 49 | 0.971502 | 31.82 |  |  | 0.58 |
| Gambia | West | 192 | 40 | 0.776416 | 58.38 |  | 33.56 | 0.13 |
| Ghana | West | 262 | 50 | 0.976977 | 8.44 | 0.5 | 38.37 | 0.38 |
| Guinea | West | 286 | 47 | 0.932592 | 2.5 |  |  |  |
| Guinea-Bissau | West | 296 | 49 | 0.971811 | 34.15 |  |  |  |
| Kenya | East | 278 | 48 | 0.957513 | 13.19 | 2.97 | 225.03 | 0.79 |
| Lesotho | South | 102 | 25 | 0.491619 | 9.98 |  | 22.83 | 0.05 |
| Liberia | West | 248 | 46 | 0.906444 | 5.42 |  |  |  |
| Libya | North | 192 | 48 | 0.955689 | 3.02 |  |  |  |
| Madagascar | East | 268 | 50 | 0.979794 | 1.08 | 0.24 | 24.7 | 0.01 |
| Malawi | South | 246 | 47 | 0.938645 | 17.52 | 0.17 | 48.27 |  |
| Mali | West | 240 | 33 | 0.652119 | 9.45 |  | 30.79 | 0.31 |
| Mauritania | North | 124 | 31 | 0.607008 | 2.33 |  |  |  |
| Mauritius | East | 248 | 47 | 0.938645 | 26.91 | 1.58 | 181.83 | 0.18 |
| Morocco | North | 156 | 47 | 0.938738 | 5.41 | 6.72 | 1068.96 | 0.71 |
| Mozambique | South | 96 | 25 | 0.491619 | 3.36 | 0.52 | 41.48 | 0.34 |
| Namibia | South | 180 | 45 | 0.902957 | 2.82 |  | 143.32 | 0.34 |
| Niger | West | 316 | 49 | 0.971811 | 1.64 |  | 7.42 |  |
| Nigeria | West | 250 | 47 | 0.942412 | 1.84 | 0.27 | 38.77 | 0.22 |
| Rwanda | East | 278 | 50 | 0.982478 | 8.14 | 0.17 | 12.35 |  |
| Saharawi Arab Democratic Republic | North | 12 | 6 | 0.116395 |  |  |  |  |
| Sao Tome-and-Principe | Central | 104 | 31 | 0.595243 | 10 |  |  |  |
| Senegal | West | 290 | 49 | 0.971811 | 6.68 |  | 549.32 | 0.75 |
| Seychelles | East | 194 | 45 | 0.908722 | 31.69 |  | 146.49 | 0.22 |
| Sierra Leone | West | 294 | 50 | 0.976977 | 5.27 |  |  |  |
| Somalia | East | 192 | 48 | 0.943198 | 0.98 |  |  |  |
| South Africa | South | 142 | 42 | 0.852552 | 77.5 | 49.68 | 473.12 | 0.8 |
| South Sudan | East | 184 | 44 | 0.898647 | 0.16 |  |  |  |
| Sudan | East | 254 | 51 | 1 | 1.79 | 7.18 |  | 0.3 |
| Tanzania | East | 264 | 49 | 0.967471 | 7.79 | 0.02 | 18.34 | 0.53 |
| Togo | West | 316 | 49 | 0.971811 | 2.1 |  | 31.77 | 0.27 |
| Tunisia | North | 192 | 48 | 0.955689 | 37.36 | 20.61 | 1964.97 | 0.6 |
| Uganda | East | 288 | 48 | 0.957513 | 15.69 | 0.39 | 26.47 | 0.17 |
| Zambia | South | 246 | 47 | 0.938645 | 15.79 | 0.84 | 40.97 | 0.28 |
| Zimbabwe | South | 246 | 47 | 0.938645 | 11.76 | 0.5 | 88.72 |  |
